# Progress Toward a Portable Artificial Lung: A Systematic Review

**DOI:** 10.1111/aor.70230

**Published:** 2026-08-27

**Authors:** Maja Munk Svendsen, Clara Kjær Hendriksen, Lars Folkersen, Peter Juhl‐Olsen, Leila Louise Benhassen, Søren Nielsen Skov

**Affiliations:** ^1^ Department of Cardiothoracic and Vascular Surgery Aarhus University Hospital Aarhus Denmark; ^2^ Department of Clinical Medicine, Faculty of Health Aarhus University Aarhus Denmark; ^3^ Department of Anesthesia and Intensive Care Aarhus University Hospital Aarhus Denmark

**Keywords:** artificial lung, chronic respiratory disease, ECLS therapy, extracorporeal membrane oxygenation, lung transplantation, mobilization, respiratory failure

## Abstract

**Background:**

Chronic respiratory diseases are the third leading global cause of death. As lung transplantation is a definitive treatment for highly selected candidates, extracorporeal membrane oxygenation (ECMO) can be a life‐sustaining therapy. Standard ECMO therapy immobilizes the patient, which increases morbidity and mortality. There is a need for a portable venovenous (VV)‐ECMO device that allows hospital ambulation and ultimately home use.

**Aim:**

This systematic review aims to identify clinically utilized and preclinical portable respiratory assist devices and describe their efficiency, progress, and innovative design features.

**Method:**

A comprehensive systematic search was conducted in six databases: PubMed, Cochrane, EMBASE, Inspect Engineering Village, ScienceDirect, and *Journal of Heart and Lung Transplantation* until July 25, 2025. A total of 562 articles were screened, and 16 were included.

**Results:**

Eight portable respiratory assist devices were identified. Among these, the CE‐marked MobyBox and the FDA‐approved Breethe OXY‐1 system are in clinical use. Two devices, the APL and the PAAL, are no longer in active development but have each laid the foundation for the Breethe OXY‐1 system and the ModELAS device. Additionally, implementation of the PAL machine was not completed but led to the development of the double‐lumen cannula. The remaining three portable ECMO devices are still undergoing preclinical testing.

**Conclusion:**

Several different portable ECMO devices have been developed, demonstrating substantial progress toward portable extracorporeal support. However, optimized anticoagulation strategies and safe mobilization require further investigation before widespread clinical adaptation.

**Trial Registration:**

PROSPERO registration number: CRD420251051796

## Graphical abstract

Eight portable respiratory assist devices were identified. The CE‐marked MobyBox and the FDA‐approved Breethe OXY‐1 system are in clinical use. Each device is substantially minimized to allow ambulation, but further work is required to address challenges related to anticoagulant management and safe mobilization. Created in BioRender. Svendsen, A. (2026) https://BioRender.com/7dhw26d.

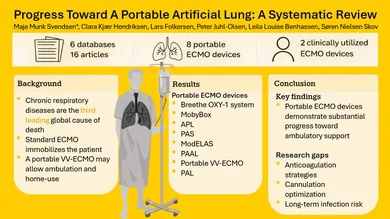

## 1 Introduction

Chronic respiratory diseases are the third leading cause of mortality worldwide [1]. Respiratory insufficiency causes hypoxia and hypercapnia accompanied by dyspnea, respiratory acidosis, and recurrent airway infections. Lung transplantation constitutes a definitive therapeutic option for carefully selected candidates; however, its application is constrained by limited organ availability and prolonged waiting times. Moreover, a substantial proportion of patients are deemed ineligible for transplantation due to advanced age or other comorbidities [2]. Extracorporeal membrane oxygenation (ECMO) treatment is a strategy to provide temporary respiratory support in cases of severe respiratory failure. In venovenous (VV) ECMO, both the inflow and outflow cannulas are positioned within the venous circulation, thereby supporting native pulmonary gas exchange without direct cardiac assistance. In contrast, venoarterial (VA) ECMO delivers both respiratory and hemodynamic support by withdrawing blood from the venous system and returning oxygenated blood to the arterial circulation. Currently, ECMO therapy necessitates admission to an intensive care unit (ICU), where patients are most often sedated and immobilized. Prolonged immobility is associated with skeletal muscle atrophy, pulmonary infections, impaired postoperative recovery, and increased morbidity and mortality [3]. Consequently, the development of a portable respiratory assist device is of considerable clinical importance, as such systems could facilitate early mobilization, enhance clearance of infections, and enable structured rehabilitation including the restoration of muscle mass following acute respiratory illness. The development of long‐term home‐based ECMO systems represents a pivotal goal, providing either a bridge to transplantation, bridge to recovery, or bridge to destination. The present study aims to systematically identify recent improvements and ongoing developments of portable ECMO devices, irrespective of their regulatory status and clinical utilization. Particular emphasis will be placed on technological strategies designed to enhance device compactness and portability. To our knowledge, this constitutes the first systematic review that identifies and describes portable respiratory ECMO devices.

## 2 Method

### 2.1 Search Methods and Data Collection

A comprehensive systematic literature search was conducted across six electronic databases: PubMed, Cochrane, Embase, Inspect Engineering Village, ScienceDirect, and the *Journal of Heart and Lung Transplantation* (JHLT). The search strategy was initially generated for PubMed and subsequently adapted to the specific syntax rules of the other databases. The primary search terms were (ECMO OR artificial lung) AND (mobile OR wearable OR ambulatory) AND (respiratory failure OR lung transplantation) and the complete search strategy can be found in Table S1. The reviewers included all relevant primary literature, irrespective of language or publication year. In addition, gray literature and reference lists of included studies were screened manually to identify further eligible studies. This systematic review was conducted in accordance with the PRISMA guidelines (Figure 1). A designed data extraction form was developed to systematically collect study characteristics including author, year of publication, country of origin, study design, machine of relevance, and a brief study description. Furthermore, articles were screened for device‐specific technical parameters, including clinical indication, battery duration, weight, O_2_ transfer rate, and blood flow capacity. If studies reported varying values for these characteristics, data from the most recent publication were included.

**FIGURE 1:**
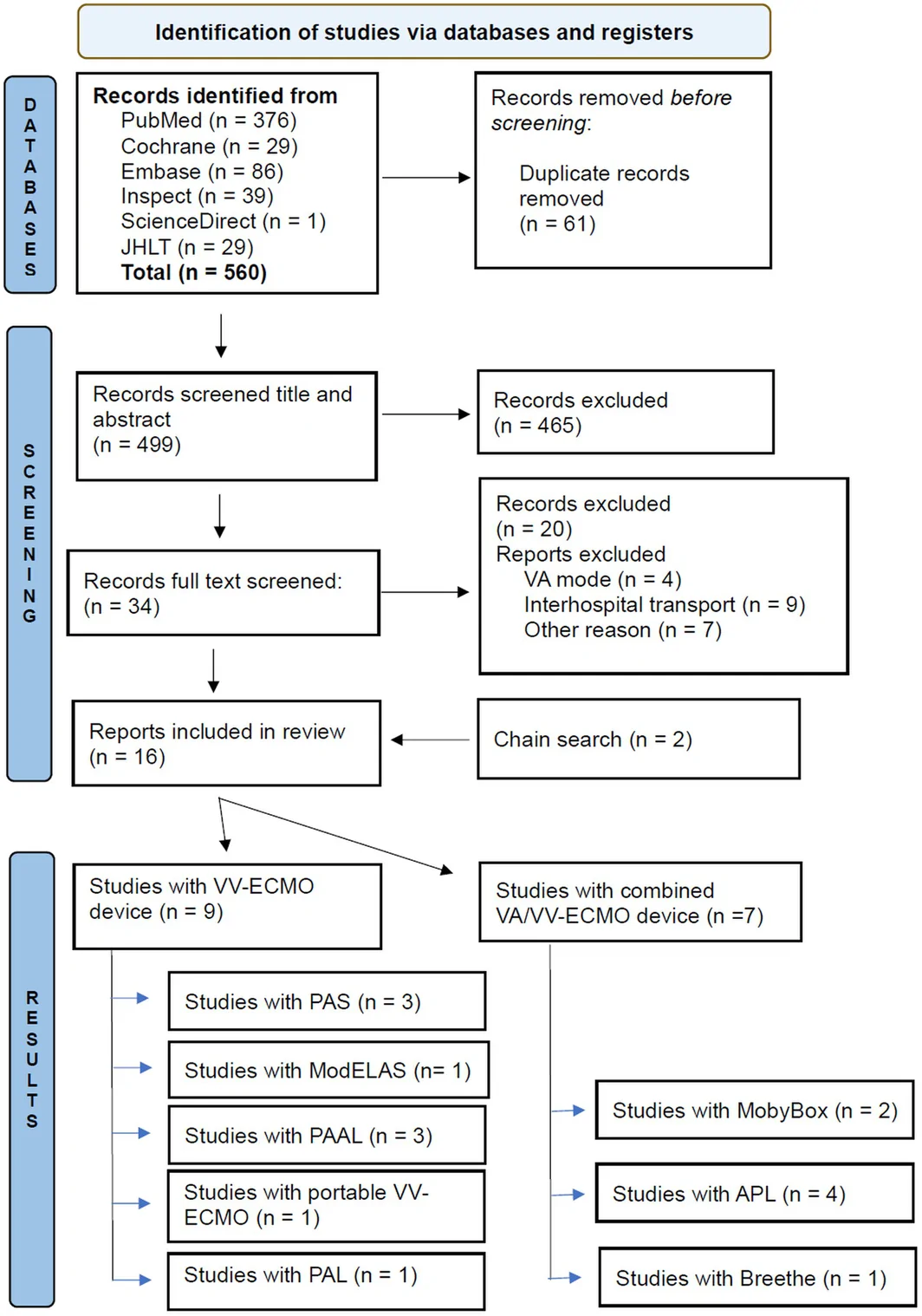
PRISMA flowchart.

### 2.2 Inclusion and Exclusion Criteria

The literature search was conducted independently by two reviewers (M.M.S. and C.K.H.), who screened titles and abstracts of all retrieved records. A total of 34 articles were deemed eligible for full‐text screening. Covidence (Veritas Health Innovation, Melbourne, Australia) was used as a tool to facilitate and streamline the screening process.

The predefined inclusion and exclusion criteria are summarized in Table 1. Studies describing ECMO devices with either isolated VV or combined VV/VA modes were eligible for inclusion (Tables 2 and 3). The most frequently excluded articles comprised reports on portable VA ECMO devices, mobile ECMO machines for interhospital transport, artificial lungs for intracorporeal placement, and portable extracorporeal carbon dioxide removal (ECCO2R) devices.

**TABLE 1:** Inclusion and exclusion criteria.

Inclusion criteria	Exclusion criteria
VV or combined VV/VA ECMO device Intended for pulmonary disease Expected or current ambulatory use Article integrates aspects of mobility, transportation, ambulation, or home use	VA ECMO and ECCO2R systems Awake standard ECMO or bedbound physiotherapy Mobile ECMO as interhospital transportation Neonatal and pediatric ECMO devices The intervention is a vehicle platform of a standard ECMO system, no efforts have been undertaken to reduce the size of the ECMO device

Abbreviations: ECCO2R, extracorporeal carbon dioxide removal; ECMO, extracorporeal membrane oxygenation.

**TABLE 2:** Included studies describing novel ECMO devices with isolated VV mode of function.

First author	Year	Location	Study type	Machine	Model, duration & findings
Roberts K. G. [4]	2023	USA	Pilot study	PAS	Seven‐ and fourteen‐days in vivo study in an ovine model (*n* = 2). The study demonstrated safe ambulation, a blood flow rate of 2 L/min, significant clot formation, brief hemolysis and a drop in platelets.
Hong Y. [5]	2024	USA	Comparative study	PAS	12‐h in vivo study in an ovine model (*n* = 15) with three interventional groups of different pumps: Axial flow pump (AFP), HeartAssist 5 (control) and Abbott Pedimag. The study demonstrated equivalent hemocompatibility among the pumps, with significantly lower hemolysis when using the AFP at start‐up.
Hong Y. [6]	2025	USA	Preclinical trial	PAS	5‐day in vivo study in an ovine model (*n* = 8) with two interventional groups: Axial flow pump (AFP) and the Abbott Pedimag (control). The study showed a significant drop in platelets with the Abbott Pedimag on day 3, stable gas exchange and minimal clot formation.
Orizondo R. A. [7]	2021	USA	Preclinical trial	ModELAS	One‐month in vivo study in an ovine model (*n* = 19) testing three different design types of the ModELAS. A single device thrombosis occurred. The study proved safe long‐term support.
Madhani S. P. [8]	2017	USA	Preclinical trial	PAAL	In vitro study of three oxygenator fiber bundles. The study proved that oxygenation efficiency was increased when the fiber bundle diameter was decreased and the length was increased.
Madhani S. P. [9]	2017	USA	Preclinical trial	PAAL	Combined in vitro and 6‐h in vivo study in an ovine model (*n* = 4). The study proved efficient pump capacity, oxygen saturation and low hemolysis.
Madhani S. P. [10]	2019	USA	Preclinical trial	PAAL	5‐day in vivo study in an ovine model (*n* = 5). The study had two non‐device related terminations, stable hemodynamics, and efficient oxygenation.
Strudthoff L. [11]	2022	Germany	Feasibility Study	Portable VV ECMO	Combined in vitro and 8‐h in vivo study in an ovine model (*n* = 6). The device was tested in a respiratory failure setting with reverse Trendelenburg position. It demonstrated stable vital parameters for most of the stages.
Zhou X. [12]	2012	USA	Preclinical trial	PAL	24‐days ambulatory in vivo study in an ovine model (*n* = 11). The study demonstrated stable hemodynamics and ambulatory long‐term respiratory support.

Abbreviations: ModELAS, Modular Extracorporeal Lung Assist System; PAAL, Paracorporeal Ambulatory Assist Lung; PAL, Paracorporeal Assist Lung; PAS, Pulmonary Assist System.

**TABLE 3:** Included studies describing novel ECMO devices with combined VV and VA functions.

First author	Year	Location	Study type	Machine	Model, duration & findings
Han D. [13]	2022	USA	Preclinical trial	Abiomed Breethe OXY‐1 system	30‐day *vivo* study in an ovine model (*n* = 1) testing the PLU device in the Breethe OXY‐1 system. The study proved a maximal oxygen transfer of 336.5 mL/min with a 4.3 L/min flow rate and an O_2_ saturation of 99.8%.
Wu Z. J. [14]	2012	USA	Preclinical study	APL	30‐day in vivo study in an ovine model (*n* = 9). Five animals made it to study termination. The four early terminations were due to mechanical failures. The study proved stable oxygen transfer, normal end‐organ function, and no major thrombi formation.
Zhang T. [15]	2012	USA	Preclinical study	APL	Combined in vitro and 6‐h in vivo study in an ovine model (*n* = 4). The study proved stable hemodynamics, saturation, and oxygen transfer with no infarcts or thrombi.
Zhou K. [16]	2013	USA	Preclinical study	APL	30‐day in vivo study in an ovine model (*n* = 8) with two interventional groups: five sheep were supported by the APL device, and three sheep were used as a sham group. All animals made it to study termination, and the APL showed successful biocompatibility with no gross thrombi formation.
Liu Y. [17]	2014	USA	Preclinical study	APL	Acute in vivo study in an ovine model (*n* = 7). The study demonstrated stable hemodynamics and blood pressure. CVP, RV ejection fraction, RV pressure and RV end‐diastolic dimensions decreased when APL flow reached 2 L/min.
Karagiannidis C. [18]	2020	Germany	Evaluation study	MobyBox	7‐day in vivo study in an ovine model (*n* = 6) supported by the MobyBox device in its VV mode of function. The study demonstrated physiological efficacy without thrombi and hemolysis.
Kau M. [19]	2022	Germany	Retrospective case series	MobyBox	Clinical outcome after MobyBox treatment of seven patients with COVID‐19‐related ARDS for 2–19 days. Three patients died and four patients improved. The MobyBox proved to be safe and efficient in a respiratory failure setting.

Abbreviations: APL, Artificial Pump Lung; ARDS, acute respiratory distress syndrome; CVP, central venous pressure; PLU, Pump‐Lung Unit; RV, right ventricle.

### 2.3 Risk of Bias Assessment

Two reviewers (M.M.S. and C.K.H.) independently assessed the quality of the included studies using the Systematic Review Centre for Laboratory Animal Experimentation (SYRCLE) risk of bias tool. The complete application of the tool can be found in Table S2. Any discrepancies between reviewers were resolved by joint reassessment and consensus. Articles with a high risk of bias were excluded. A modified SYRCLE was used for the Madhani et al. [8] in vitro study. The search identified a single retrospective case series (Kau et al. [19]) which was addressed using the Joanna Briggs Institute (JBI) critical appraisal checklist and the outcome can be found in Table S3.

## 3 Results

### 3.1 Study Selection

The search identified 560 articles, 61 of which were duplicates and two were included from the chain‐search. A total of 34 articles were selected for full‐text screening, and 16 met all the eligibility criteria. Due to heterogeneity among the studies, no pooling was conducted in this systematic review. The last search was performed on July 25, 2025.

Eight portable respiratory assist devices were identified (Table 4). Two devices are clinically utilized; The CE‐marked MobyBox and the FDA‐approved Breethe OXY‐1 system. The Artificial Pump‐lung (APL), the Paracorporeal Artificial Lung (PAL), and the Paracorporeal Ambulatory Assist Lung (PAAL) are no longer in active development but served as foundation for further innovations. The Modular Extracorporeal Lung Assist System (ModELAS) and the portable VV ECMO are still undergoing preclinical testing.

**TABLE 4:** Device characteristics for the portable VV and VV/VA ECMO devices.

Machine	Indication	Battery	Weight	O_2_ transfer	Blood flow	Cannulation	Adverse events
Breethe OXY‐1 system [13, 20]	VA/VV mode	3.5 h	1.3 kg^a^	400 mL/min	1–5 L/min	Medtronic DLP 32Fr	Thrombi formation Controller malfunction in MAUDE report System failure in MAUDE report
APL [14, 15, 16]	VA/VV mode	1.5–2 h [13]	13.06 kg	180 mL/min	1–5 L/min	Medtronic DLP 32Fr	Device failure
MobyBox [18]	VA/VV mode	Power‐independent	2.8 kg^b^	> 300 mL/min	1–5 L/min	Novaport Twin 24Fr	Cannula site bleeding Thrombi formation Reversible platelet drop and cessation of spontaneous breathing in the in vivo study
PAS [4, 5, 6]	VV mode	4–12 h	3.2 kg	> 100 mL/min	1–3 L/min	28Fr Crescent DLC	Thrombi formation Hemolysis Platelet drop
ModELAS [7]	VV mode	—	1 kg^c^	143 mL/min	1–4 L/min	Avalon Elite Bicaval DLC 27Fr	Thrombi formation Cannula fracture Bleeding Increased motor torque
PAAL [8, 9, 10]	VV mode	—	1.8 kg	> 180 mL/min	1–4 L/min	Avalon Elite Bicaval DLC 26Fr	Thrombi formation Kinked cannula and cannula site bleeding Air‐leak due to broken stop‐cock
Portable VV‐ ECMO [11]	VV mode	—	1.24 kg	> 160 mL/min	1–4 L/min	Novaport twin 24Fr	Blood flow below 3 L/min due to small cannula size
PAL [12]	VV mode	—	—	220 mL/min	1–4.4 L/min	Avalon Elite Bicaval DLC 27Fr	Thrombi formation Bleeding Gas exchanger failure

Abbreviations: DLC, Double Lumen Cannula; DLP, Double Lumen perfusion; MAUDE, Manufacturer and User Facility Device Experience; VA, Venoarterial; VV, Venovenous.

^a^ Without console weight of 22.5 kg.

^b^ Without controller unit weight of 2.15 kg.

^c^ Without controller unit weight.

### 3.2 Abiomed Breethe OXY‐1 System and Artificial Pump‐Lung

The Abiomed Breethe OXY‐1 system (Abiomed, a Johnson & Johnson Medtech company, Danvers, MA, USA) is designed to provide both pulmonary and cardiac support. It offers a maximum battery duration of 3.5 h, a blood flow rate of 1–5 L/min, and an oxygen transfer capacity of up to 400 mL/min [13]. The device is comprised of an integrated pump‐lung unit (PLU) incorporating disposable elements for long‐term use and a wheeled console to regulate pump speed and sweep gas delivery (Figure 2). The system uses a single pivot bearing centrifugal pump with a hollow‐fiber membrane oxygenator. The PLU device can be worn in a shoulder‐strap or hung on the adjacent console. A distinguishing characteristic of this system is its ability to provide respiratory support without the need of pressurized medical oxygen, thereby enhancing compactness and portability. Independence from pressurized oxygen is achieved through an integrated oxygen concentrator that delivers 98% oxygen concentrated from ambient air [13, 23]. Specifically, the Breethe OXY‐1 system operates in two distinct modes: an internal gas (portable) mode utilizing ambient air, and an external oxygen (stationary) mode, supplied via piped‐in oxygen or a gas cylinder (Figure 3). In portable mode, the system can deliver up to 3 h of support without external power or a gas source [24]. Han et al. [13] evaluated the PLU of the Breethe OXY‐1 system in an ovine model for up to 30 days with device exchanges enabling survival of up to 120 days. During this period, the Breethe OXY‐1 system reached a maximal oxygen transfer of 336.5 mL/min and a blood flow rate of 4.3 L/min, while maintaining stable hemodynamics [13].

**FIGURE 2:**
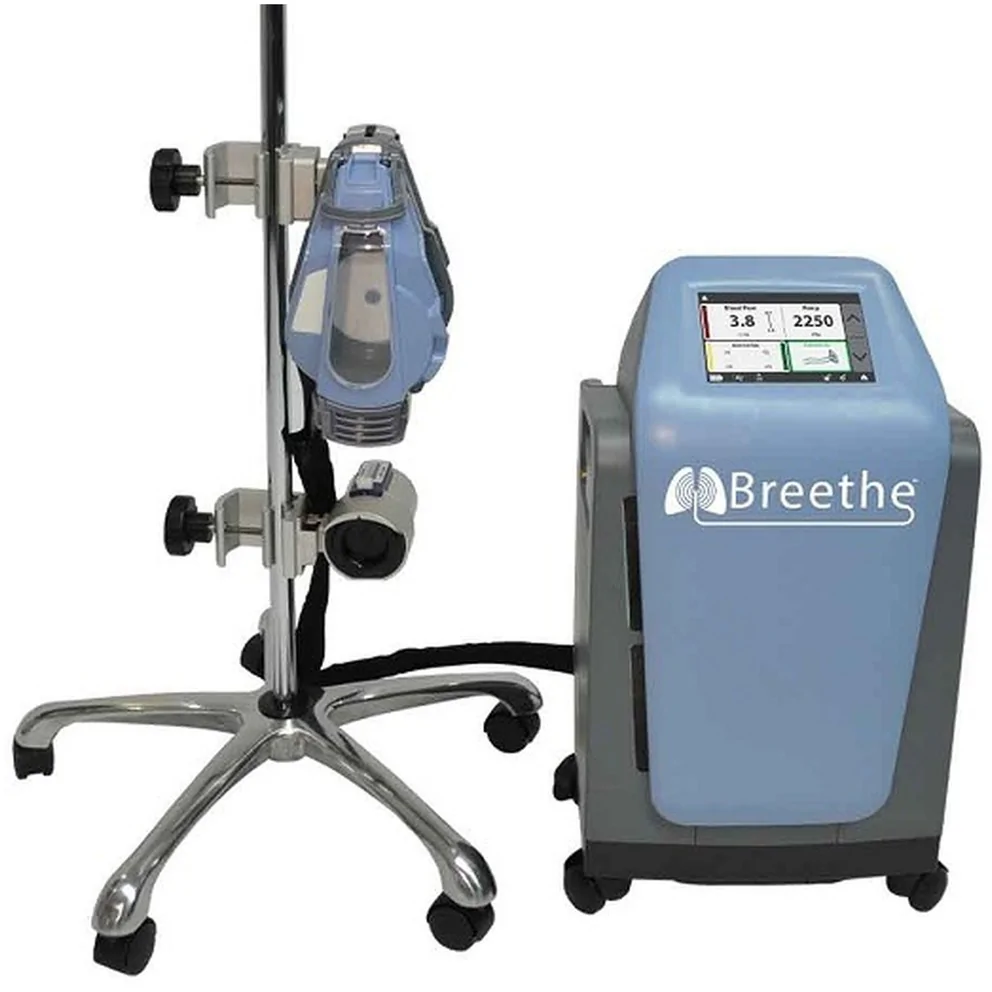
Abiomed Breethe OXY‐1 system. *Source:* Johnson & Johnson MedTech Heart Recovery [21, 22].

**FIGURE 3:**
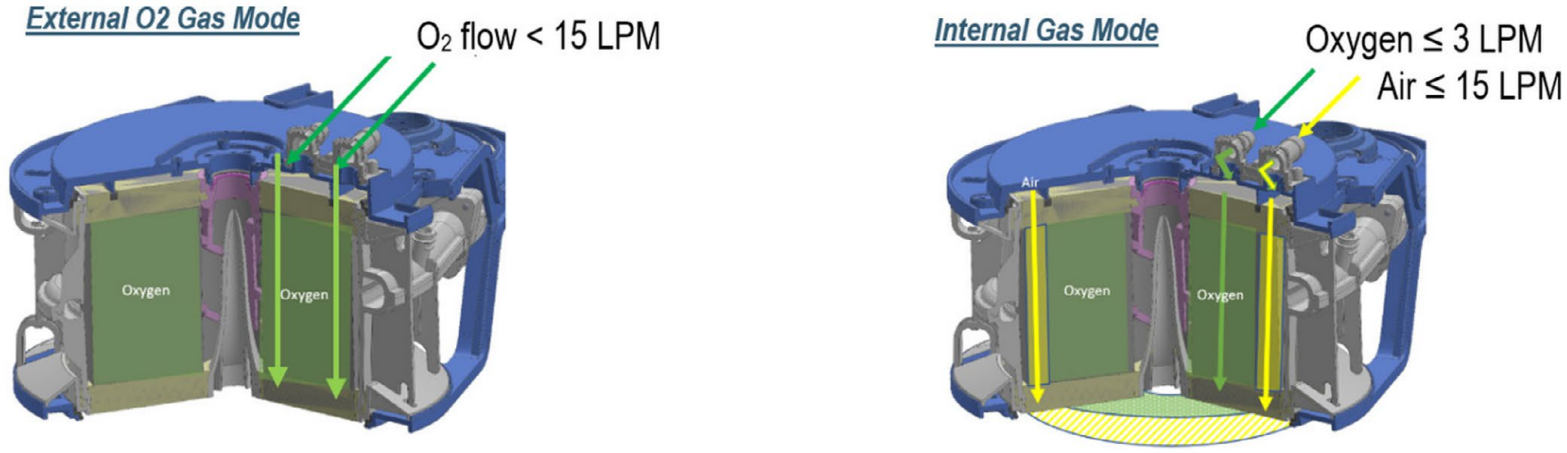
Abiomed Breethe OXY‐1 oxygenator. The oxygenator is separated into an inner core and an outer surface. When the device runs in the stationary mode (left), oxygen is supplied in both the inner core and outer surface. The O_2_ flow can reach 15 L/min in this mode. When the device runs in portable mode (right), ambient air passes through the outer surface to sweep CO_2_ and is concentrated by an oxygen concentrator to 93%, which is passed through the inner core for blood oxygenation [24]. *Source:* Johnson & Johnson MedTech Heart Recovery [21].

The Breethe OXY‐1 system was FDA‐approved in 2020 for 6 h of use and has since then been successfully utilized for patient treatment in multiple centers in the United States. The Abiomed Breethe OXY‐1 device is indicated for long‐term (> 6 h) respiratory or cardiopulmonary support in adults with acute respiratory or cardiopulmonary failure. It is intended for use in patients whom conventional treatment options have failed and continued clinical deterioration or imminent risk of death is anticipated. Contraindications include vascular access problems, irreversible organ injuries, or conditions deemed unsuitable for hemodynamic stabilization. The device is compatible with commercially available cannulas fitting 3/8‐in. PVC tubing, while the cannulation strategy is determined by the physician based on the individual patient's clinical condition [24]. Table 4 illustrates adverse events during trials and clinical utilization. Han et al. reported the formation of HFM thrombi measuring < 1 mm. In addition, the device has been described in four FDA MAUDE reports. Two involve controller malfunction [25, 26], one documents an undetermined system failure [27], and one reports patient hypothermia, bradycardia and hypo‐coagulability [28]. The currently marketed “Abiomed Breethe OXY‐1 system” represents an updated version of the prior designed APL [13]; consequently the APL will not be addressed separately in this review.

### 3.3 MobyBox

The MobyBox (Hemovent GmbH, Aachen, Germany) is a German‐developed device currently approved for up to 14 days of use in either VV or VA ECMO configurations. Notably, it is pneumatically driven and power‐independent. The system incorporates a two‐chamber pump, in which the pneumatic pressure from the gas initially powers the pump and subsequently continues to the oxygenator, thereby serving a dual function. The device comprises a console and a 2.8 kg patient unit containing both the pump and an oxygenator (Figure 4). The MobyBox provides an oxygen transfer exceeding 300 mL/min and supports a blood‐flow of up to 5 L/min. It consumes 4 L of pure oxygen per minute, providing approximately 100 min of therapy with a 200 L bar gas tank. Karagiannidis et al. [18] tested the MobyBox in a 7‐day ovine in vivo study, and found that the system demonstrated physiological efficacy without evidence of thrombus formation on visual inspection, and all animals survived until study termination. The MobyBox obtained European CE certification in 2019 and was successfully used in man for the first time in December 2020 during the second wave of COVID‐19. Seven patients were admitted to the center with COVID‐19‐related ARDS and received pulmonary support with VV‐ECMO. Six patients were initially supported with conventional ECMO systems before being transitioned to the MobyBox, whereas one patient was treated with the MobyBox from initiation. One patient developed significant cannula‐site bleeding, and another had a thrombotic event, however, the authors did not attribute these to device malfunction. No device failures or complications were observed, and no disadvantages were identified compared with the initially used conventional VV ECMO systems. Two patients exceeded the upper limit use of 14 days without any duration‐related complications. The authors suggested that the MobyBox may aid in out‐of‐hospital emergency settings and areas with limited infrastructure due to its power‐independence [19]. As indications, the MobyBox is intended to provide extracorporeal cardiopulmonary support for patients with established or impending respiratory, cardiac, or cardiorespiratory failure. During operation, the system must be used in conjunction with an external vital‐signs monitoring device that includes, at minimum, an oxygen saturation alarm function. The intended patient population comprises adults aged 16 years or older who meet the indications of use and have no contraindications. Contraindications to use the MobyBox device include coagulation disorders, vessel diseases, or the need for long term support for more than 30 days. The cannulation strategy depends on the clinical indication. The MOBYBOX is compatible with standard single‐ and double‐lumen cannulas designed for ECLS applications, provided they are equipped with a 3/8‐in. tubing connector. VA‐ and VV‐applications include femoral‐femoral, femoral‐jugular, and double‐lumen jugular configurations [30].

**FIGURE 4:**
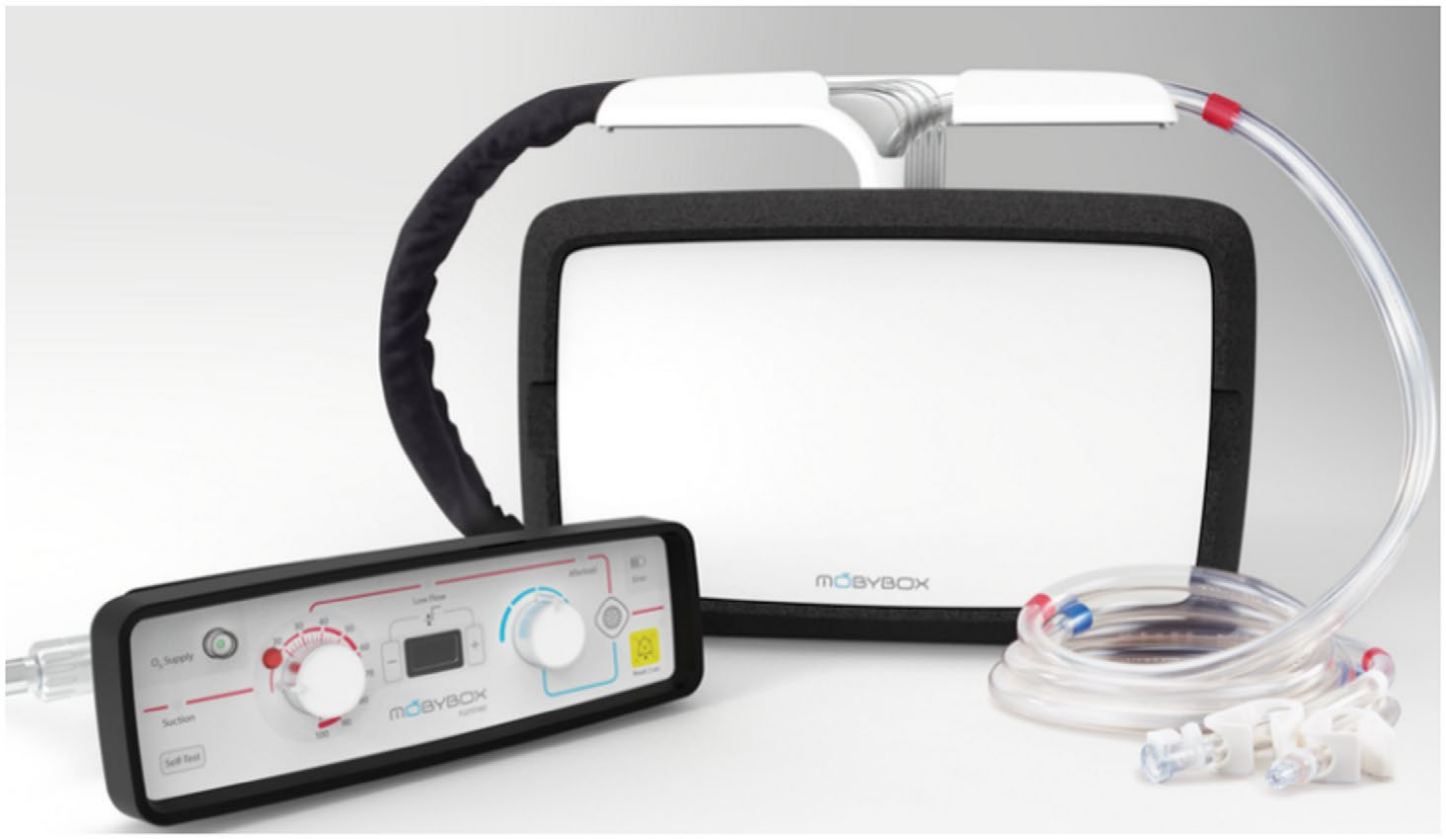
The MobyBox control master unit and patient unit. *Source:* Hemovent.com/produkte/mobybox [29].

### 3.4 Pulmonary Assist System

The Pulmonary Assist System (PAS) device is a novel, preclinical VV‐ECMO system intended to provide long‐term ambulatory respiratory support for patients with chronic lung disease, either in a hospital setting or potentially in outpatient care [5]. The device has an operational blood flow rate of 1–3 L/min and an oxygen transfer exceeding 100 mL/min. It incorporates a hollow‐fiber gas‐exchanger (PAS‐GE) and a battery‐powered axial flow pump (AFP), with a total weight of 3.2 kg allowing transport in a small backpack. The novel key design feature is the non‐integrated configuration separating the lightweight AFP from the artificial lung component. This modular approach addresses a common limitation in ECMO therapy—oxygenator thrombosis—resulting from the large thrombogenic surface area of hollow‐fiber membranes. Because oxygenators require more frequent replacement than pumps, separation of these components facilitates more efficient and cost‐effective exchange [6]. The PAS has been evaluated in sheep for up to 14 days, with animals ambulating on the final study day to demonstrate feasibility of mobilization. As adverse events, they found significant clot formation, brief hemolysis, and a drop in platelets [4]. The AFP was also compared with two established control pumps (HeartAssist 5 and Abbott Pedimag), in a 12‐h ovine study, demonstrating comparable hemocompatibility and reduced hemolysis compared with controls [5]. The PAS device is yet to be tested clinically in humans.

### 3.5 Modular Extracorporeal Lung Assist System and Paracorporeal Ambulatory Assist Lung

The ModELAS is a portable VV ECMO device designed to provide long‐term respiratory support for up to 1–3 months. Weighing approximately 1 kg, it incorporates a custom centrifugal pump and a hollow‐fiber membrane (HFM) oxygenator. Its compactness derives from a small HFM bundle with a surface area of 0.65 m^2^—the smallest surface area among the reviewed systems—thereby reducing thrombogenic surface area. For comparison, the Breethe OXY‐1 system features a surface area of 2.4 m^2^ [7]. The device supports a maximal blood flow rate of up to 4 L/min. It was evaluated in an in vivo ovine model in a 28–30 day trial [7]. Of 19 animals, 10 were supported for the full duration. Study termination occurred due to cannula fracture (*n* = 2), bleeding (*n* = 3), bearing thrombosis (*n* = 3), and increased motor torque (*n* = 1). Cannulation‐related and perioperative complications were acknowledged as significant limitations. In this study, blood flow did not reach the rated flow of 4 L/min, resulting in perfusion‐limited oxygen transfer; therefore, definitive conclusions regarding fiber bundle performance could not be drawn. The PAAL preceded ModELAS and was evaluated in vitro [8], and was further tested in an acute in vivo setting [10]. Notably, the in vitro study demonstrated flow rates up to a maximal 4 L/min, contrary to the in vivo limitations observed in the ModELAS in vivo trial. Although the PAAL device is no longer under active development, it provided a technological foundation for ModELAS, which has not yet undergone clinical testing.

### 3.6 Portable VV‐ECMO

The portable VV‐ECMO device weighs 1.24 kg and has an operational blood flow rate of 1–4 L/min. Similar to the Breethe OXY‐1 system, it blends ambient air with pressurized medical oxygen. The device was evaluated by Strudthoff et al. [11], and a human prototype is available. Strudthoff et al. [11] evaluated the device in an ovine model of combined hypercapnic and hypoxic respiratory failure. The included devices of the other studies all used healthy animals, which limited the transferability of the results to patients with respiratory failure. The device was tested for 8 h with different F_i_O_2_s and respiratory frequencies in a supine position and in reversed Trendelenburg to imitate an upright patient. Hemodynamic stability was maintained in five of six animals across all settings; however, blood flow only reached 3 L/min, below the nominal functional limit. The authors suggested that a different cannula could have been used [11].

### 3.7 Paracorporeal Artificial Lung

Ambulation with the PAL device is facilitated by the Avalon Elite double‐lumen cannula, formerly known as the PAL‐groups' patented Wang‐Zwische double‐lumen cannula. The article was published in 2012 when the APL device was also under development. In contrast to the APL device, PAL did not require open thoracic surgery for device implantation. At the time of publication, the commercially available double‐lumen cannula was only used for pediatric patients, in contrast to the Avalon Elite double‐lumen cannula, which could also be used for adult patients. The study reported blood flow rates of 2 L/min and three cannula‐related terminations due to kinking or dislocation. The authors expected the DLC to run up to 4.4 L/min. The Avalon Elite double‐lumen cannula has since become commercially available and is now widely used in VV‐ECMO practice [12].

## 4 Discussion

In summary, eight portable ECMO devices have been evaluated between 2012 and 2025, leading to clinical implementation of two devices: the Breethe OXY‐1 system and the MobyBox. Although no longer under active development, the APL and PAAL provided the technological foundation for the subsequent development of the Breethe OXY‐1 system and the ModELAS. Portable ECMO systems are specifically engineered to mitigate and withstand complications associated with ECMO. The oxygenator constitutes a major artificial thrombogenic surface and must balance competing design demands: it must be sufficiently large to provide adequate oxygen delivery while remaining compact enough to permit mobility and minimize thrombogenicity. The disposable PLU of the Breethe OXY‐1 system permits selective replacement during long‐term support in cases of biofouling or thrombosis. In addition, the transparent PLU enables visual inspection for thrombus formation and blood‐flow alterations [23]. Han et al. [13] demonstrated effective respiratory support with no clinically significant thrombus formation or bleeding complications. Similarly, the oxygenator of the MobyBox is engineered to optimize blood washout, limit blood stagnation, and eliminate low‐flow regions. To achieve this, the corners of the oxygenator are sealed, and the blood flow is directed helically into the fiber bundle. The previously reported thrombotic event during clinical application was attributed to a preceding arterial catheterization and was considered unrelated to the device itself [18, 19]. The ModELAS device incorporates the smallest oxygenator surface area among the reviewed systems of only 0.65 m^2^, while maintaining adequate oxygenation. However, two cases of decreased fiber bundle permeability were reported, both associated with upper or lower bearing thrombosis, leading to early termination on Day 22 and 23. The authors suggested that transient thrombi were formed at the bearing and subsequently embolized to the fiber bundle, although overall long‐term gas exchange remained stable [7].

Following demonstrations of device efficacy and functionality, evaluation of mobility represents a relevant subsequent focus of investigation. Some studies incorporated mobility assessments alongside their primary endpoints. Roberts et al. [4] evaluated the PAS device in an ambulatory setting, with sheep walking on a tether and subsequently roaming freely while supported by the device, thereby demonstrating feasibility of safe ambulation. Additionally, weight reduction of the PAS device to approximately 2 kg is planned to enhance ambulation [5]. In contrast, acute trials involving devices such as the portable VV‐ECMO and the PAAL did not evaluate ambulation directly. However, the portable VV‐ECMO device was assessed in a setting imitating an upright or semi‐recumbent patient by operating the device in a reverse Trendelenburg position. Given that the efficacy and functionality of the portable VV‐ECMO devices have been thoroughly tested and shown to be satisfactory, future investigations should prioritize systematic evaluation of ambulatory performance and feasibility in out‐of‐hospital environments.

An ideal portable respiratory assist device should be compact and lightweight to facilitate ambulation. A body‐worn design, such as a backpack or shoulder bag, promotes patient independence by allowing both hands to remain free during ambulation and activities, but it requires the device to only weigh a few kilograms. A device designed to be transported alongside the patient, like a suitcase configuration, may accommodate a greater weight before portability becomes substantially compromised. From a physiological perspective, reductions in size and weight should not come at the expense of device performance; A VV ECMO device should preferably be capable of delivering blood flow rates of around 2–3 L/min, whereas a VA ECMO device should ideally provide even higher blood flow rates. The oxygenator should ensure adequate oxygen transfer (DO_2_) and carbon dioxide elimination while maintaining low thrombogenicity and allowing straightforward replacement during prolonged support.

Adequate carbon dioxide elimination depends on the sweep gas supply. In conventional ICU ECMO systems, sweep gas is typically supplied as wall‐mounted medical oxygen, which is suitable in settings where patient mobility is not required. Currently, pressurized medical oxygen remains the most widely used sweep gas source for portable ECMO devices. However, it increases device weight, reduces portability, and requires periodic cylinder replacement. An alternative is an integrated oxygen concentrator, which could eliminate the need for pressurized medical oxygen. However, such a solution would increase power consumption which may limit its ability to provide high continuous flow rates, which can reach 10 L/min. As described in the results section, many of the abovementioned innovations have been incorporated into the devices included in this review. However, a technological gap remains. For example, patient mobility introduces abrupt physiological fluctuations in oxygen consumption and metabolic demand that are not typically encountered during sedated ICU‐based ECMO support. An ideal portable respiratory assist device should therefore incorporate an adaptive control algorithm capable of automatically adjusting blood pump speed and sweep gas flow in response to changing physiological demands.

A portable ECMO device should optimally also incorporate a reliable battery system supporting prolonged ambulation, while allowing recharging, straightforward replacement, and redundant power sources to ensure uninterrupted operation. Similar challenges have been successfully addressed in other battery‐operated devices, such as the left ventricular assist devices (LVADs). These devices are powered by portable battery systems with hot‐swappable batteries and facilitate mobilization. LVAD therapy has evolved from an exclusively hospital‐based treatment to one that can be safely managed in outpatient settings. While their purpose is fundamentally different from ECMO devices, they demonstrate that long‐term ambulatory extracorporeal support is feasible when reliable portable power systems are available [31].

The concept of mobilizing ECMO patients is also seen in awake ECMO therapy, which is becoming more accepted and utilized [32]. Awake ECMO is ECMO support in awake, spontaneously breathing patients. Awake ECMO enables patients to participate in physiotherapy, including closed kinetic exercises, standing with or without support, and short‐distance ambulation. However, these activities require close supervision by a specialized multidisciplinary team, typically consisting of an intensivist, nurse, physiotherapist, respiratory therapist, and perfusionist [33]. Although both awake ECMO and portable ECMO systems aim to facilitate patient mobility, they differ in their intended clinical application. Awake ECMO remains an in‐hospital treatment strategy based on conventional ECMO platforms, where mobilization is performed under continuous supervision by healthcare professionals. In contrast, portable ECMO systems have been developed with the long‐term endpoint of out‐of‐hospital care, with the goal of enabling greater patient independence and improving quality of life in individuals requiring prolonged respiratory support. The portable ECMO devices should be regarded as complementary therapy to the awake ECMO and standard ECMO support. As exemplified by the MobyBox, these systems are currently used as such and evaluated in hospital settings during the early stages of clinical implementation before their potential role in out‐of‐hospital care can be established.

Device mobility represents one of several relevant issues that remain to be addressed before portable ECMO systems can be safely implemented outside the hospital setting. The cannulation site poses a significant risk of infection due to its close association with the bloodstream; even minor infections may rapidly progress to a life‐threatening septic condition. Further investigation is warranted regarding prophylactic antibiotic strategies, optimal subcutaneous cannula length, and standardized infection surveillance protocols. Additional unresolved issues include the selection of an appropriate anticoagulant regimen and response protocols for device malfunction. Cannulation strategies, in terms of adequate sizing and positioning, are especially important to provide sufficient support while minimizing the wound opening with concurrent risks of bleeding and infection. Overall, clinical trial data on device performance remain limited and should be prioritized in future research.

### 4.1 Study Limitations

Publication bias may have influenced the findings of this review. Although the inclusion of gray literature was intended to mitigate this risk, unpublished relevant data may exist which could not be accounted for. Heterogeneity among the included studies constituted an additional limitation and may have impeded direct comparability between devices; however, some systems were not tested at maximal capacity, whereas others were perfusion‐limited due to suboptimal cannula sizing. Therefore, maximal oxygen transfer rates may be higher than those reported in this review. Device evaluated at maximal capacity may therefore appear comparatively advantageous. Similarly, reported device weights must be interpreted cautiously. Some investigators reported the weight of the entire ECMO assembly, including oxygen source, battery, and consoles, whilst others reported the weight of the novel pump‐lung unit. Therefore, the weight of the devices should be compared with caution. Furthermore, most in vivo investigations were conducted in healthy animal models. To more accurately reflect physiological performance in clinical practice, evaluation in models of respiratory failure would be preferable. Such a setting was implemented by Strudthoff et al. [11] when assessing their portable VV‐ECMO under varying fractions of inspired oxygen levels and respiratory frequencies. The Breethe OXY‐1 system and the MobyBox have already demonstrated clinical efficacy in patients with COVID‐19‐associated acute respiratory distress syndrome.

### 4.2 Risk of Bias

The reliability of the findings is inherently dependent on the quality of the studies included. Overall, included studies performed favorably when evaluated with the SYRCLE risk of bias tool. The majority demonstrated a low risk of detection bias as they conducted random outcome assessment, ensuring objective and unbiased evaluation of results. Additionally, studies adequately addressed attrition bias by including all animals in the final analysis, appropriately reporting and handling missing data. Two studies were assessed as having a high risk of selection bias due to non‐randomized grouping, potentially affecting unmeasured baseline characteristics. However, in studies involving small sample sizes, non‐randomized allocation may represent a pragmatic methodological choice, as the statistical benefits of randomization and stratification increase with larger cohorts. Five studies were assessed as having a high risk of funding bias or conflicts of interest. While such bias constitutes a methodological concern, it also represents a practical and technical limitation, as industrial expertise and commercial involvement often are necessary to enable technological development and implementation in the field of portable ECMO devices.

## 5 Conclusion

Eight portable respiratory assist devices were identified in this review. Among the three devices with VV/VA support, only the Breethe OXY‐1 and MobyBox systems are currently in clinical use. The Breethe OXY‐1 system has received FDA approval for 6 h use and has been implemented clinically. Its novel design is strategically focused on ambulation with the possibility of 3 h ambient‐air based oxygen and battery time. The novel designed MobyBox also focuses on portability through complete power independence but is limited by the need for an external oxygen supply. It has obtained CE certification for 14 days of use and published data substantiate successful clinical feasibility.

In addition, five portable VV‐ECMO devices have been evaluated. Although PAL device was not fully implemented, its development contributed substantially to the introduction of an integrated double‐lumen cannula which has significantly influenced contemporary VV‐ECMO practice. Currently, three portable VV‐ECMO devices, the ModELAS, PAS, and the portable VV‐ECMO device, are currently under active development. While each has achieved notably progress in device miniaturization further research is required to address challenges related to anticoagulant management, infection prevention, and safe mobilization prior to widespread adoption.

## Author Contributions

M.M.S.: conceptualization; data analysis; data collection; drafting article; risk of bias. C.K.H.: data analysis; data collection, critical revision of article, approval of article. L.F.: conceptualization; critical revision of article; approval of article. P.J.O.: conceptualization; critical revision of article; approval of article. L.L.B.: conceptualization; data analysis; data collection; critical revision of article; approval of article. S.N.S.: conceptualization, data analysis, data collection, critical revision of article, approval of article.

## Conflicts of Interest

The authors declare no conflicts of interest.

## Supporting information


**Table S1:** Search strategy generated for PubMed and adjusted to the additional databases.
**Table S2:** SYRCLE risk of bias assessment.
**Table S3:** JBI critical appraisal checklist.

## Data Availability

The data that supports the findings of this study are available in the Supporting Information of this article.
