# Supplementary material for: Progress Toward a Portable Artificial Lung: A Systematic Review
Source: Artif Organs. 2026 Aug 27;50(11):1520–31. doi: 10.1111/aor.70230 (PMC13720653; doi:10.1111/aor.70230)
Supplement: Supplementary file 1 [file AOR-50-1520-s001.docx]

#### Supplementary Table 1: Search strategy generated for PubMed and adjusted to the additional databases

| **PubMed/MEDLINE**  *Filter: [all fields]*  **Cochrane**  *Filter: [title, abstract or Author keywords]*  **JHLT**  *Filter: [title, abstract or Author keywords]* | *(ECMO OR “respiratory support” OR "venovenous ECMO" OR "extracorporeal membrane oxygenation" OR ECLS OR VV-ECMO OR "artificial lung") AND (mobility OR ambulatory OR miniaturized OR mobilization OR mobile OR wearable OR compact OR portable OR lightweight) AND ("acute respiratory distress syndrome" OR ARDS OR "chronic obstructive pulmonary lung disease" OR "lung transplantation" OR COPD OR "lung failure" OR "interstitial lung disease" OR "restrictive lung disease" OR "respiratory failure" OR "artificial lung" OR "artificial lungs") OR ("Breethe OXY-1 system" OR "Abiomed breethe extracorporeal membrane oxygenation system" OR Mobybox) NOT (neonatal OR pediatric)* |
| --- | --- |
| **Embase**  *Filter:* Database: EMBASE | #1*(ECMO OR “respiratory support” OR "venovenous ECMO" OR "extracorporeal membrane oxygenation" OR ECLS OR VV-ECMO OR "artificial lung")/br.*  AND  #2*(mobility OR ambulatory OR miniaturized OR mobilization OR mobile OR wearable OR compact OR portable OR lightweight).ti.*  AND  #3*("acute respiratory distress syndrome" OR ARDS OR "chronic obstructive pulmonary lung disease" OR "lung transplantation" OR COPD OR "lung failure" OR "interstitial lung disease" OR "restrictive lung disease" OR "respiratory failure" OR "artificial lung" OR "artificial lungs").ti.*  *OR*  *#4("Breethe OXY-1 system" OR "Abiomed breethe extracorporeal membrane oxygenation system" OR Mobybox).ti.*  NOT  #5*(neonatal OR pediatric).ti,ab,kw* |
| **Inspect Engineering Village** | #1*(ECMO OR “respiratory support” OR "venovenous ECMO" OR "extracorporeal membrane oxygenation" OR ECLS OR VV-ECMO OR "artificial lung"). All fields*  AND  #2*(mobility OR ambulatory OR miniaturized OR mobilization OR mobile OR wearable OR compact OR portable OR lightweight).ti.*  AND  #3*("acute respiratory distress syndrome" OR ARDS OR "chronic obstructive pulmonary lung disease" OR "lung transplantation" OR COPD OR "lung failure" OR "interstitial lung disease" OR "restrictive lung disease" OR "respiratory failure" OR "artificial lung" OR "artificial lungs").su,ti,ab,*  *OR*  *#4("Breethe OXY-1 system" OR "Abiomed breethe extracorporeal membrane oxygenation system" OR Mobybox). su,ti,ab,*  NOT  #5*(neonatal OR pediatric). su,ti,ab,* |
| **ScienceDirect** | #1*(Ambulatory OR mobile OR mobility) AND (respiratory failure). ti,ab,kw.*  #2*(Extracorporeal membrane oxygenation system).ti.* |
| **Chain-search** | Detected Wu Z. J. (2012) and Zhang T. (2012) from Zhou K. (2013) |

#### Supplementary Table 2: SYRCLE risk of bias assessment

|  | Selection bias - Sequence generation | Selection bias - Baseline characteristics | Selection bias - Allocation concealment | Performance bias - Random housing | Performance bias - Blinding | Detection bias - Random outcome assessment | Detection bias - blinding | Attrition bias - Incomplete outcome data | Reporting bias - Incomplete outcome data | Other - Conflict of interest or funding bias |
| --- | --- | --- | --- | --- | --- | --- | --- | --- | --- | --- |
| Roberts K. G. |  |  |  |  |  |  |  |  |  |  |
| Hong Y. (2024) |  |  |  |  |  |  |  |  |  |  |
| Hong Y. (2025) |  |  |  |  |  |  |  |  |  |  |
| Orizando R. A. |  |  |  |  |  |  |  |  |  |  |
| Madhani (b) |  |  |  |  |  |  |  |  |  |  |
| Madhani (2019) |  |  |  |  |  |  |  |  |  |  |
| Strudthoff L. |  |  |  |  |  |  |  |  |  |  |
| Zhou K. |  |  |  |  |  |  |  |  |  |  |
| Wu Z. J. |  |  |  |  |  |  |  |  |  |  |
| Zhang T. |  |  |  |  |  |  |  |  |  |  |
| Karagiannidis C |  |  |  |  |  |  |  |  |  |  |
| Liu Y. |  |  |  |  |  |  |  |  |  |  |
| Han D. |  |  |  |  |  |  |  |  |  |  |
| Zhou X. |  |  |  |  |  |  |  |  |  |  |
| Madhani S. (a) |  |  |  |  |  |  |  |  |  |  |

| Selection bias - Sequence generation | |  | |  |  |  |  |  |  | |  |  |  |  |  |  |  |  |
| --- | --- | --- | --- | --- | --- | --- | --- | --- | --- | --- | --- | --- | --- | --- | --- | --- | --- | --- |
|  | | | | | | | | | | | | | | | |  |  |  |
| Selection bias - Baseline characteristics | |  | |  |  |  |  |  |  | |  |  |  |  |  |  |  |  |
|  | | | | | | | | | | | | | | | |  |  |  |
| Selection bias - Allocation concealment | |  | |  |  |  |  |  |  | |  |  |  |  |  |  |  |  |
|  | | | | | | | | | | | | | | | |  |  |  |
| Performance bias - Random housing | |  | |  |  |  |  |  |  | |  |  |  |  |  |  |  |  |
|  | | | | | | | | | | | | | | | |  |  |  |
| Performance bias - Blinding | |  | |  |  |  |  |  |  | |  |  |  |  |  |  |  |  |
|  | | | | | | | | | | | | | | | |  |  |  |
| Detection bias - Random outcome assessment | |  | |  |  |  |  |  |  | |  |  |  |  |  |  |  |  |
|  | | | | | | | | | | | | | | | |  |  |  |
| Detection bias - Blinding | |  | |  |  |  |  |  |  | |  |  |  |  |  |  |  |  |
|  | | | | | | | | | | | | | | | |  |  |  |
| Attrition bias - Incomplete outcome data | |  | |  |  |  |  |  |  | |  |  |  |  |  |  |  |  |
|  | | | | | | | | | | | | | | | |  |  |  |
| Reporting bias - Selective outcome reporting | |  | |  |  |  |  |  |  | |  |  |  |  |  |  |  |  |
|  | | | | | | | | | | | | | | | |  |  |  |
| Other - Conflict of interest or Funding bias | |  | |  |  |  |  |  |  | |  |  |  |  |  |  |  |  |
|  | | | | | | | | | | | | | | | |  |  |  |
| Low risk of bias | Unclear risk of bias | | High risk of bias | | | | | | | Not Applicable | | | | | |  |  |  |

#### Supplementary Table 3: JBI critical appraisal checklist

|  | Were there clear criteria for inclusion in the case series? | Was the condition measured in a standard, reliable way for all participants included in the case series? | Were valid methods used for identification of the condition for all participants included in the case series? | Did the case series have consecutive inclusion of participants? | Did the case series have complete inclusion of participants? | Was there clear reporting of the demographics of the participants in the study? | Was there clear reporting of clinical information of the participants? | Were the outcomes or follow up results of cases clearly reported? | Was there clear reporting of the presenting site(s)/clininc(s) demographic information? | Was statistical analysis appropriate? |
| --- | --- | --- | --- | --- | --- | --- | --- | --- | --- | --- |
| Kau M. (2022) |  |  |  |  |  |  |  |  |  |  |
